# Gut Bacteria Regulate the Pathogenesis of Huntington’s Disease in *Drosophila* Model

**DOI:** 10.3389/fnins.2022.902205

**Published:** 2022-06-02

**Authors:** Anjalika Chongtham, Jung Hyun Yoo, Theodore M. Chin, Ngozi D. Akingbesote, Ainul Huda, J. Lawrence Marsh, Ali Khoshnan

**Affiliations:** ^1^Biology and Bioengineering, California Institute of Technology (Caltech), Pasadena, CA, United States; ^2^Developmental and Cell Biology, University of California, Irvine, Irvine, CA, United States

**Keywords:** Huntington’s disease, microbiota, gut-brain, neurodegeneration, crocin (PubChem CID: 5281233)

## Abstract

Changes in the composition of gut microbiota are implicated in the pathogenesis of several neurodegenerative disorders. Here, we investigated whether gut bacteria affect the progression of Huntington’s disease (HD) in transgenic *Drosophila melanogaster* (fruit fly) models expressing full-length or N-terminal fragments of human mutant huntingtin (HTT) protein. We find that elimination of commensal gut bacteria by antibiotics reduces the aggregation of amyloidogenic N-terminal fragments of HTT and delays the development of motor defects. Conversely, colonization of HD flies with *Escherichia coli* (*E. coli*), a known pathobiont of human gut with links to neurodegeneration and other morbidities, accelerates HTT aggregation, aggravates immobility, and shortens lifespan. Similar to antibiotics, treatment of HD flies with small compounds such as luteolin, a flavone, or crocin a beta-carotenoid, ameliorates disease phenotypes, and promotes survival. Crocin prevents colonization of *E. coli* in the gut and alters the levels of commensal bacteria, which may be linked to its protective effects. The opposing effects of *E. coli* and crocin on HTT aggregation, motor defects, and survival in transgenic *Drosophila* models support the involvement of gut-brain networks in the pathogenesis of HD.

## Introduction

Huntington’s disease (HD) is a progressive genetically inherited neurodegenerative disorder characterized by debilitating motor, psychiatric, and cognitive symptoms (Bates et al., 2015; Ghosh and Tabrizi, 2018). Expansion of a CAG repeat (>35) in exon 1 of the huntingtin (HTT) gene, which translates into an abnormal polyglutamine (polyQ) tract, is the underlying cause of HD (Huntington’s Disease Collaborative Research Group, 1993). The expanded polyQ enhances the amyloidogenic properties of HTT exon1 (HTTex1) peptide, which misfolds and forms insoluble protein assemblies in neurons (DiFiglia et al., 1997). Expansion of the polyQ repeat is a major determinant of disease onset in HD, however, multiple genetic and environmental factors may affect the development and progression of symptoms. One potential modifier is neuroinflammation exemplified by elevated levels of inflammatory microglia and TH17.1 cells in the brains of pre-manifest HD patients. An increase in the activated immune cells coincides with elevated production of inflammatory cytokines, which persists during the symptomatic stages of HD (Björkqvist et al., 2008; Politis et al., 2015; von Essen et al., 2020). Although mutant HTT has been directly implicated in the activation of inflammatory pathways, the environmental inducers of inflammation in HD remain to be investigated (Khoshnan et al., 2004; Trager et al., 2014; Khoshnan et al., 2017).

Commensal and acquired gut microorganisms are prominent sources of inflammogens implicated in neurological disorders. Within the last decade, extensive studies demonstrate that the gastrointestinal (GI) tract and its resident microbes (collectively known as microbiota, and their genomes as microbiome) regulate the nervous system physiology. For example, the gut microbiota influences neurodevelopment, neurodegeneration, neurotrophin and neurotransmitter production, neuropsychiatric and motor behaviors, and neuroinflammation (Sampson et al., 2016; Sharon et al., 2016; Morais et al., 2021). Changes in the homeostasis of gut microbiota (dysbiosis) have been linked to the pathogenesis of several neurological and neurodegenerative disorders, including, autism spectrum disorder (ASD), multiple sclerosis (MS), Alzheimer’s disease (AD), Parkinson’s disease (PD), and amyotrophic lateral sclerosis (ALS) (Hsiao et al., 2013; Sampson et al., 2016; Marizzoni et al., 2020; Takewaki et al., 2020; Zeng et al., 2020). Neuroinflammation is a prominent feature of gut dysbiosis in neurodegenerative disorders, which includes the activation of microglia and subsequent production of inflammatory cytokines (Sampson et al., 2016; Abdel-Haq et al., 2019). Notably, inflammatory bacteria such as *Enterobacteriaceae* are elevated in the gut of PD patients and their abundance correlates with the worsening of the neurological and pathological symptoms (Keshavarzian et al., 2015; Scheperjans et al., 2015; Li et al., 2017). Moreover, lipopolysaccharides (LPS), major components of gram negative bacteria, which include *Enterobacteriaceae*, have been implicated in the pathogenesis of PD (Perez-Pardo et al., 2019). Gut dysbiosis manifested by reduced alpha diversity in the bacterial communities were recently reported in a cohort of HD patients. Notably, in these studies changes in the abundance of *Eubacterium halii* and potentially other candidates coincide with altered cognition (Wasser et al., 2020). Another gut microbiome analysis of a group of HDs patient’s links blooming of the gram-negative bacteria *Bilophila* species to elevated levels of inflammatory cytokines (Du et al., 2021). In transgenic mouse models of HD expressing the neurotoxic mutant HTTex1 fragment, gut dysbiosis has been linked to weight loss, motor deficit, metabolic changes, and disruption in the intestinal epithelium (Kong et al., 2020; Stan et al., 2020; Kong et al., 2021). These promising studies highlight a potential role of gut microbiota in the pathogenesis of HD. However, the mechanisms of how a specific bacterium may affect disease manifestation remain to be investigated.

*Drosophila melanogaster* (fruit fly) is emerging as a useful model to study the impact of gut-brain interactions in the development and progression of neurological disorders (Wu et al., 2017; Douglas, 2018). Similar to mammals, gut homeostasis in *Drosophila* is regulated by the interaction of bacteria with the enteric neurons and intestinal epithelium including the enteroendocrine cells, which produce antimicrobial peptides (AMPs) involved in immunity against invasive pathogens and maintaining optimal abundance of commensal bacteria (Hanson and Lemaitre, 2020). *Drosophila* has a handful of commensal gut bacteria, which can easily be manipulated for studies on gut-brain communications (Douglas, 2018; Ankrah et al., 2021). *Lactobacillus* and *Acetobacter* species are the two most abundant bacterial genera, which regulate nutrient acquisition, growth, metabolism, immune development, and locomotion (Schretter et al., 2018; Storelli et al., 2018; Henriques et al., 2020; Yamauchi et al., 2020; Ankrah et al., 2021). The *Drosophila* models of HD display several hallmarks of disease progression including protein aggregation, motor defects, and aberrant expression of genes including those implicated in systemic inflammation (Barbaro et al., 2015; Al-Ramahi et al., 2018). The simplicity of *Drosophila* gut microbiota offers a useful platform to examine the influence of endogenous and single exogenous bacterial species on HD pathogenesis and to investigate the role of HTT in gut–brain pathways. As a first step, we explored whether elimination of gut bacteria in HD *Drosophila* models or colonization with the human pathobiont *E. coli* influences disease development. Here, we report that gut bacteria promote the aggregation of amyloidogenic N-terminal fragments of HTT, contribute to development of aberrant motor behavior and reduce the life span of female HD flies. We further provide evidence that modifying the gut environment of female HD flies ameliorates HD phenotypes. These studies and models facilitate future dissection of gut-brain interaction in HD at a molecular level and are useful for the discovery of gut-based therapeutics.

## Materials and Methods

### Fly Stocks

The huntingtin expressing transgenic *Drosophila* lines used in this study were M{UAS- HTT.ex1.Q25} (B#68414), M{UAS-HTT.ex1.Q120} (B#76352), and M{UAS-HTT.586.Q120} (B#68447), M{UAS-HTT.FL.Q25} (B#68397), M{UAS-hHTT.FL.Q120} (Barbaro et al., 2015; Chongtham et al., 2020). Human HTT 586, full-length HTT and HTTex1 (90 aa) transgenes were inserted into the same chromosomal location (51D) and in the same orientation in a common inbred host *Drosophila* line using the phiC31 targeted-insertion system (Bischof et al., 2007). The Gal4/UAS system (Brand and Perrimon, 1993) was used to express the HTT transgenes by crossing the transgenes under the control of the UAS promoter to a driver line having the yeast Gal4 transcriptional activator. The GAL4 drivers used were the pan-neuronal *elav*-Gal4 C155 driver (Bloomington Stock Number B#458) and the ubiquitous *da*-Gal4 (B#8641). Fly cultures were maintained on standard cornmeal/sugar/agar media on a 12:12 h light:dark cycle. Appropriate crosses were carried out to obtain desired progeny). Briefly, standard food vials with ten UAS-HTT males and ten *da*-Gal4 or *elav*-Gal4 driver females were allowed to mate for 2 days and then passed into new vials at 20°C. All assays used female progeny, which were collected after the eclosion and maintained at 20°C for 2–3 days until enough flies had been collected.

### Treatment of Flies With Antibiotics or Small Molecules

For drug treatment, groups of 15–20 female flies were placed in vials containing standard cornmeal/sugar/agar food alone for non-treatment control experiments or standard food mixed with 1 mg/ml of water-soluble test compounds crocin (cat# 17304 Sigma), rifaximin (cat# Y0001074, Sigma), luteolin (# L9283 Sigma), or 1% penicillin-streptomycin (cat#15140-122; Gibco). The flies were transferred to 25°C and passaged to fresh vials every 2nd or 3rd day. For treatment with live bacteria, curli producing (MC4100) and curli deficient (Δcsg) *E. coli* strains from −80°C frozen stocks were grown on YESCA (1% Casamino Acids, 0.12% yeast extract, 2% Bacto agar) agar plates at 25°C for 48 h. The *Lactobacillus rhamnosus* strain (JB-1, ATCC) from frozen stock was streaked on MRS agar (cat#OXCM0361B, Fisher) plates and grown at 37°C overnight. The *Acetobacter* stock was prepared by grinding adult flies in PBS followed by culturing of isolated bacteria on mannitol agar plates. Isolated colonies were grown in liquid manitol broth at 37°C in a bacterial shaker. The bacterial cultures were harvested by centrifugation (3000×g, 5 min), washed and suspended in PBS. The bacterial suspension (0.5 OD or 5 × 10^7^ cells) was then mixed with standard food and groups of 15–20 female progeny that had first been treated with 1% penicillin-streptomycin- supplemented food for 3–4 days, were transferred to the bacteria supplemented food. The flies were transferred to new food with the live bacteria every 2nd or 3rd day at 25°C.

To examine the effects of crocin on *E. coli* treated flies, crocin (1mg/mL) and *E. coli* (0.5 OD or 5 × 10^7^ cells) were both mixed with standard food and flies, that had been treated with 1% penicillin-streptomycin for 3–4 days, were transferred to the crocin and *E. coli* mixed food. Flies were transferred to fresh food every 2nd or 3rd day and their bacterial load and motor behavior was monitored.

### Western Blotting

At least 10 wandering third instar larvae or adult flies were lysed and homogenized in RIPA buffer (25 mM Tris-HCl pH 7.6, 150 mM NaCl, 1% NP-40, 1 mM EDTA) with protease inhibitors (Complete, Mini Protease Inhibitor Cocktail, and Roche Applied Science). The lysates were then boiled at 95°C for 5 min, and equal amounts of protein were separated by SDS/PAGE on pre-cast 4–20% polyacrylamide gradient gels (Cat# 5671094, Biorad) and transferred to immune-blot PVDF membrane (Merck cat# IPVH00010). Membranes were blocked with blocking solution (5% non-fat milk in 0.05% Tween in PBS) and incubated with primary anti-HTT antibody PHP1 (1:1,000 in blocking solution) overnight at 4°C. The blots were then treated with HRP-conjugated goat anti-mouse secondary antibody (1:10,000) diluted in blocking solution for 1 h and developed with enhanced chemiluminescent (ECL) substrate (Cat#1705060, Biorad).

To separate SDS-resistant amyloid assemblies, semi-denaturing detergent agarose gel electrophoresis (SDD-AGE) was performed (Halfmann and Lindquist, 2008) with some modifications. Fly lysates were prepared as described above and resolved by electrophoresis in SDD-AGE gels (1.5% agarose, 1X TAE, 0.1% SDS). Proteins were transferred to immune-blot PVDF membrane by overnight downward capillary action using 1X TBS. Membranes were then treated as western blots above.

### Immunostaining of Larval Brains and Guts of Adult Flies

Wandering third instar larvae were cut into anterior and posterior halves, and the anterior halves were turned inside out and placed in PBS on ice. These halves were then fixed by rocking for 30 min at RT with 4% formaldehyde made in PBST (PBS + 0.2% Triton X-100). After fixation, halves were washed three times with PBST, blocked with 5% BSA in PBT for 1 h at RT, probed overnight with primary antibody (s) at 4°C, washed, blocked again, and incubated with secondary antibody (s) for 2 h and washed again. Larval brains were then dissected out and mounted in Vectashield-DAPI medium. The primary antibodies were rat-Elav-7E8A10 anti-elav (used at 1:200 dilution in PBS; Developmental Studies Hybridoma Bank), PHP1 anti-HTT (used at 1:500 dilution in PBS; Ko et al., 2018). The secondary antibodies were Alexa Fluor 488 goat anti-mouse (green) and Alexa Fluor 568 goat anti-rat (red) (used at 1:250 dilution in blocking solution, Life Technologies).

Whole guts of adult flies were dissected out in PBS, fixed in 4% formaldehyde in PBST for 1 h, washed with PBST and incubated with blocking buffer for 1 h at RT. The guts were then probed with anti-*E. coli* monoclonal antibody (produced in house) overnight at 4°C. After washing with PBST, guts were incubated with Alexa Fluor 488 goat anti-mouse antibody diluted in blocking buffer for 2 h at RT and washed. The guts were mounted in Vectashield-DAPI medium. Images of mounted tissues were captured using a Leica Sp8 laser scanning microscope and analyzed using Leica Application Suite X (LAS X) software.

### Seeding Assay

Seeding assay was according to recent protocols published recently (Chongtham et al., 2021). Briefly, 5 μg of fly lysate from control, *E. coli* and *E. coli* (curli+) fed flies was preincubated with 0.01 μg/mL of proteinase K at 37°C for 1 h and heat inactivated at 75°C for 10 min. The proteinase K treated fly lysate was then incubated with 100 μg of total protein from human neural lysates for 4 h at 25°C with continuous agitation. Semi-denaturing detergent agarose gel electrophoresis (SDD-AGE) was performed to analyze the seeded products.

### Climbing Assay

For monitoring the locomotor ability, 10 flies were gently tapped to the bottom of a vertical glass vial (diameter, 2.2 cm), as adapted from Liu et al. (2008). The number of flies that climbed to a height of 5 cm within 10 s was recorded. The test was repeated three times each for four vials of 10 flies at 1 min intervals.

### Longevity Assay

A longevity assay was performed as described previously with slight modifications (Barbaro et al., 2015). Briefly, eighty eclosed female flies (1–3 days old) were divided into four tubes of 20 each with the indicated treatment in the figure legends and were maintained at 20°C with a 12:12 h light:dark cycle. Longevity assays in the presence of *E.coli* were performed at 25°C to accommodate bacterial growth. Progeny were collected and transferred to fresh vials with standard food ± treatment. Cultures were monitored every other day and the number of dead flies counted.

### Congo Red Staining of Bacterial Colonies and Colony Forming Unit Counting

To determine the *E. coli* (Curli-producing) load in the gut, flies were surface sterilized with 70% ethanol for 1 min and washed three times with sterile 1X PBS. Flies were then homogenized in groups of five in 500 μL of sterile 1X PBS using a motorized pestle. Microbial counts were determined by serial dilution plating of the homogenates on YESCA agar plates supplemented with 50 μg/mL of Congo Red (CR) (Sigma). The YESCA CR plates were incubated at 25°C for 2 days to induce curli production and the number of red colonies that showed curli expression were counted.

### Quantification of Dead Pupae

Four groups of 10 UAS-HTTex1-120Q males and elav-Gal4 female virgins were allowed to mate for 2 days at 20°C and passaged daily into vials with standard food supplemented with crocin (1 mg/mL) or 1% penicillin-streptomycin for a span of 4 days. Twenty days after crosses were made, all flies were emptied from the vials, and empty pupal cases and cases containing dead flies were counted to calculate the percentage of pupal survival (Hatfield et al., 2015).

#### PCR Amplification Bacteria

Flies were surface sterilized with 70% ethanol for 1 min and washed three times with sterile 1X PBS. Bacterial DNA was extracted from equal number of HTTex1 (25Qs or 103Qs) using stool DNA kit (Omega Bio-tek, Norcross, Georgia) according to the provided procedures. Bacterial DNA was amplified from 5 ng of total DNA from each batch of fly by q-pCR using the universal primer Eub340F: 5′-TCCTACGGGAGGCAGCAGT-3′ and Eub781R: 5′-GGACTACCAGGGTATCTAATCCTGTT-3′) (Nadkarni et al., 2002) in a 7300 real-time PCR system. Data were analyzed comparatively by the formula 2^(–ΔΔCT)^.

#### Construction of pLenti6-csgA-6x his Plasmid

*E. coli* genomic DNA was isolated from ∼1 mL of overnight culture using QIAamp DNA Mini Kit (QIAGEN), following the manufacturer’s protocol. C-terminally hexahistidine-tagged CsgA without N-terminal SEC secretion signal sequence (Evans et al., 2015) was amplified via standard PCR method using the following primers: csgA F: 5′-TCAAGGGAATTCACCATGGGTGTTGTTCCTCAGTACGG-3′, csgA R: 5′-TCAACGGGATCCCTAGTGATGATGGTGGTGA TGGTACTGATGAGCGGTCGCGTTG-3′. Thermal cycling program used for the PCR is as follows: 94°C, 5 min → 33x [94°C, 30 s → 58°C, 30 s → 72°C, 30 s] → 72°C, 7 min → 4°C. The resulting PCR amplicon was gel-purified using QIAquick Gel Extraction kit (QIAGEN) and then assembled into pLenti6/V5-D-TOPO vector (Invitrogen) following the manufacturer’s protocol. The cloned plasmid was subsequently transformed into One Shot TOP10 chemically competent *E. coli* (Invitrogen) and spread on LB + Ampicillin (100 μg/mL) agar plate for selection. Several plasmid clones were isolated using QIAprep Spin Miniprep kit (QIAGEN) and then screened via agarose gel electrophoresis after digesting them with *Eco*RI and *Bam*HI. The correct clone was sequenced using CMV-F primer (5′-CGCAAATGGGCGGTAGGCGTG-3′) and named pLenti6-V5-D-TOPO:csgA-6x His.

#### Co-transfection of HTTex1 Q103-EGFP and csgA Plasmids in HEK293 Cells

HEK293 cells were seeded in a 6-well plate with approximately 25% density and let grow overnight at 37°C, 5% CO_2_. The cells were then transfected using calcium phosphate transfection method. In brief, 0.1 μg Lenti-HTTex1 Q103-EGFP plasmid and differential amounts of Lenti–csgA-6x His plasmid (0, 0.2, or 0.3 μg), were added in each snap-cap tube. Beta-galactosidase cDNA cloned in similar vector was used as control. The total amount of DNA in each transfection mixture was normalized using an empty plasmid backbone. 2 M CaCl_2_ was subsequently added (0.12 M final concentration), and the volume of mixture was brought up to 100 μL with ddH_2_O. 100 μL 2x HBS was added next dropwise and part of solution was squirted into the rest by pipetting for aeration. Entire volume of each transfection mixture was added to the cells and incubation was done overnight at 37°C, 5% CO_2_. The transfected cells were subjected to widefield fluorescence microscopy for quantifying HTTex1 Q103-EGFP aggregates and then harvested for SDS-PAGE and western blotting analyses for HTTex1 Q103-EGFP (probed with PHP2 antibody, 1:1,000 dilution) and CsgA-6x His (probed with mouse anti-His antibody, from Thermo Fisher, 1:5,000 dilution).

### 16S Sequencing of Gut Bacteria

Female flies were harvested on the indicated days post-eclosion, gently disinfected in 70% ethanol and three subsequent rinses in PBS to remove any external bacteria and stored immediately at −80°C. Similar samples for different batches of flies were mixed and shipped to the sequencing facility at Zymo Research Irvine, CA, United States. Briefly, bacterial DNA was extracted using ZymoBIOMICS-96 MagBead DNA kit (Zymo Research Irvine, CA, United States). The DNA samples were prepared for targeted sequencing with the Quick-16S NGS library Prep kit and 16S primer set V3-V4 (Zymo Research Irvine, CA, United States). The final library was sequenced on Ilumina MiSeq with a V3 reagent Kit (600 cycles). The sequencing was performed with 10% PhiX Spike-in.

### Bioinformatics Analysis of Bacteria

Unique amplicon sequences were inferred from raw reads using the Dada2 pipeline (Callahan et al., 2016). Chimeric sequences were also removed with the Dada2 pipeline. Taxonomy assignment was performed using Uclust from Qiime v.1.9.1. Taxonomy was assigned with the Zymo Research Database, a 16S database that is internally designed and curated, as reference. Composition visualization, alpha-diversity, and beta-diversity analyses were performed with Qiime v.1.9.1 (Caporaso et al., 2010). If applicable, taxa that have significant abundance among different groups were identified by LEfSe (Segata et al., 2011) using default settings. Other analyses such as heatmaps, Taxa2SV_deomposer, and PCoA plots were performed with internal scripts.

### Absolute Abundance Quantification

Quantitative real-time PCR was set up with a standard curve. The standard curve was made with plasmid DNA containing one copy of the 16S gene and one copy of the fungal ITS2 region prepared in 10-fold serial dilutions. The primers used were the same as those used in Targeted Library Preparation. The equation generated by the plasmid DNA standard curve was used to calculate the number of gene copies in the reaction for each sample. The PCR input volume (2 μl) was used to calculate the number of gene copies per microliter in each DNA sample. The number of genome copies per microliter DNA sample was calculated by dividing the gene copy number by an assumed number of gene copies per genome. The value used for 16S copies per genome is 4. The value used for ITS copies per genome is 200. The amount of DNA per microliter DNA sample was calculated using an assumed genome size of 4.64 × 10^6^ bp, the genome size of *Escherichia coli*, for 16S samples, or an assumed genome size of 1.20 × 10^7^ bp, the genome size of *Saccharomyces cerevisiae*, for ITS samples. This calculation is shown below: Calculated Total DNA = Calculated Total Genome Copies × Assumed Genome Size (4.64 × 106 bp) × Average Molecular Weight of a DNA bp (660 g/mole/bp) ÷ Avogadro’s Number (6.022 × 1,023/mole).

### Statistical Analysis

Error bars show Standard Error of the Mean (SEM = standard deviation/square root of *n*). Statistical significance was established using analysis of variance (ANOVA) on Prism software (GraphPad). A one-way ANOVA was performed to analyze the effect of bacterial or drug treatment on motor behavior and survival. A two-way ANOVA was performed to analyze the effect of bacterial or drug treatment, and the timeline of treatment on motor behavior and survival. A two-way ANOVA was performed to check the effect of genotype (WT and mutant HTT expression) and age of flies (days post eclosion) on microbial load of flies. A two-way ANOVA was also performed to investigate the impact of bacterial treatment or gender, and genotype (Da > Gal4, WT-HTT, and mutant HTT) on motor behavior. The ANOVA results are summarized in Supplementary Table 1 (* = *P* < 0.05, ^**^ = *P* < 0.01, and ^***^ = *P* < 0.001).

## Results

### Gut Bacteria Promote the Aggregation of Amyloidogenic HTT Fragments in *Drosophila*

**FIGURE 1:**
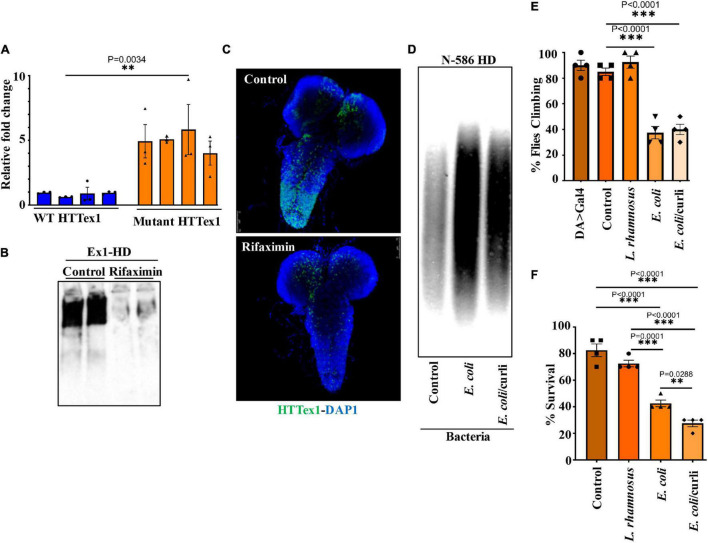
Microbiota modulate HTT aggregation, locomotor behavior, and lifespan of *Drosophila* HD models. **(A)** qPCR amplification of total bacteria from DNA extracted from different batches of HTTex1-expressing offspring. Data are shown as fold change over the value for one batch of WT HTTex1. **(B)** Western blot detection of mutant HTTex1 aggregates in the lysates of untreated or rifaximin-treated Ex1-HD larvae, using anti-HTT (PHP1) antibody. Each lane represents 10 pooled larvae (*N* = 10) isolated from a different fly vial. **(C)** Representative confocal images of brains (*N* = 3) from the Ex1-HD larvae treated with rifaximin and immunolabelled with anti-HTT (PHP1, green). DAPI (blue) was used to stain the nuclei. **(D)** SDD-AGE and WB analysis of lysates (50 μg each) derived from female N-586 HD flies untreated (control), or colonized with *E. coli* or *E. coli* expressing curli. PHP1 antibody was used to detect HTT aggregates. For each condition 10 larvae were pooled together for analysis (*N* = 10). **(E)** Female N-586 HD flies were fed curli-producing/deficient *E. coli or L. rhamnosus.* A climbing assay was performed at day 20 after eclosion as described in M&M. Data are reported as mean ± SEM and were analyzed by one-way ANOVA with Tukey’s *post hoc* test. ^***^p < 0.001, *n* = 4 groups of 10 flies. **(F)** The percentage of flies surviving at day 20 was calculated and plotted for each experimental condition. Data are represented as mean ± SEM and were analyzed by one-way ANOVA with Tukey’s *post hoc* test. ^***^p < 0.001; ^**^p < 0.01, *n* = 4 groups of 10 flies.

Transgenic *Drosophila* expressing HTTex1 (120Qs) under the control of the pan-neuronal driver *elav*-Gal4 (Ex1-HD) display gut dysbiosis exemplified by elevated levels of total bacteria when compared to a line expressing WT HTTex1 (25Qs) (Figure 1A and Supplementary Figures 1A,B). To examine whether gut bacteria influence the aggregation of mutant HTTex1, we generated larvae of the Ex1-HD line in the presence of a gut-specific antibiotic, rifaximin, to eliminate bacteria (Supplementary Figure 1C) and examined for the accumulation of aggregates by Western blots (WBs) and immunohistochemistry (IHC). Rifaximin treatment significantly reduces the levels of HTTex1 aggregates in the nervous system of Ex1-HD larvae (Figures 1B,C). Given that HTTex1 is selectively expressed in neurons, these findings suggest that gut bacteria may alter neuronal physiology and pathways which regulate the misfolding and aggregation of HTTex1. We then asked whether gut bacteria with links to neurodegeneration in humans may have a similar effect. *Escherichia coli* (*E. coli)*, a gut pathobiont, has been implicated in the pathogenesis of PD. In particular, colonization of transgenic mice expressing human α-synuclein, or normal rats with an *E. coli* strain expressing the functional bacterial amyloids curli accelerates the aggregation of α-synuclein in the brain, induces neuroinflammation and worsens motor symptoms (Chen et al., 2016; Sampson et al., 2020). We used a *Drosophila* line ubiquitously expressing the N-terminal 586 amino acids (AA) fragment of mutant HTT (120Qs) (N-586 HD) to explore the impact of *E. coli* on aggregation. Protein aggregates in this line accumulate much slower than in the Ex1-HD line thus, making it ideal for these assays (Barbaro et al., 2015). N-586 HD larvae were generated on food containing an *E. coli* strain expressing curli or an isogenic mutant where the operon for curli has been deleted (Reichhardt et al., 2015). We find that colonization with either strain accelerates the aggregation of N-586 HTT fragment (Figure 1D). Although we do not observe any selective effects of curli on HTT proteostasis in the N-586 female HD flies, recombinant curli promotes the aggregation of mutant HTTex1-EGFP when co-expressed in HEK-293 tissue culture cells (Supplementary Figure 2). A likely explanation is that direct interaction of curli with mutant HTT may be essential to induce misfolding, whereas other aggregation-promoting components of *E. coli* common to both strains may function by indirect pathways. Collectively, these findings identify gut bacteria as modifiers of HTT aggregation in fruit fly models of HD. We also explored whether *E. coli* induces protein aggregation in transgenic female flies ubiquitously expressing full-length mutant HTT (120Qs) (FL-HD flies). Using various aggregate-specific antibodies (Ko et al., 2018; Chongtham et al., 2021), we did not detect any HTT aggregates in HD flies treated with or without *E. coli* (Supplementary Figure 3). However, we cannot rule out the formation of unstable oligomers or novel conformations, which may not bind to antibodies used in these studies.

### Gut Bacteria Contribute to Immobility and Death of Female Flies Expressing Mutant HTT

**FIGURE 2:**
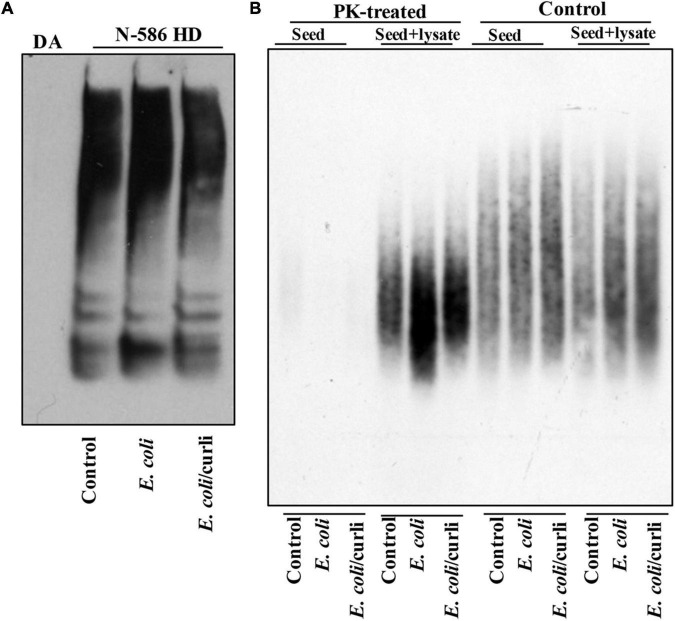
*E. coli* enhances the seeding activity of mutant HTT assemblies in female N-586 HD flies. **(A)** SDS-PAGE analysis of brain lysates followed by WBs of ∼15-day old non-transgenic (DA) or N-586 HD flies treated with *E. coli* probed with anti-HTT PHP1. Details are provided in the section “Materials and Methods.” **(B)** SDD-AGE of equal amounts of seeds from lysates in part A added to 50 μg of human neuronal lysates before or after treatment with proteinase-K (PK) (0.5 μg/ml of PK at RT for 30 min and subsequently inactivated by heating at 75°C (Chongtham et al., 2020). Seeds mixed with neuronal lysates were incubated at RT for 4 h and subsequently examined by SDD-AGE. Products were probed with PHP1 antibody.

Abnormal motor behavior is a cardinal symptom of HD. Gut bacteria have been implicated in the locomotion of *Drosophila* (Schretter et al., 2018). Given that *E. coli* promotes HTT aggregation, we asked whether it may alter the mobility of HD flies. The N-586 HD flies do not develop mutant HTT-mediated motor defects at a young age (Barbaro et al., 2015), however, colonization of female flies with *E. coli* significantly impairs their climbing ability, whereas *Lactobacillus rhamnosus* (JB-1 strain) *(L. rhamnosus)*, a gram-positive human probiotic has no effects (Figure 1E). Moreover, *E. coli* does not alter the mobility of non-transgenic female flies or those expressing WT HTT (Supplementary Figure 4A). These findings suggest that *E. coli* may promote the toxicity of the N-586 HTT fragment, which manifests as motor defects in the transgenic flies. *E. coli* also shortens the lifespan of N-586 HD flies; by day 20∼50% of the flies die and the rest remain immobile and expire within few days (Figure 1F). Curli-producing *E. coli* appears more toxic than the isogenic mutant strain indicating that the bacterial amyloids may affect the mortality of N586-HD flies independent of HTT aggregation and locomotor defects (Figure 1F). Seeding of mutant HTT species isolated from the brains of HD *Drosophila* models has been linked to disease progression and toxicity (Ast et al., 2018). We recently reported that seeding-competent HTTex1 species produce neurotoxic assemblies in human neurons and neuronal lysates. The seeding competency of HTT species isolated from mouse models of HD is induced by proteinase-K (PK) treatment (Chongtham et al., 2021). To determine whether *E. coli* colonization enhances the seeding competency of mutant HTT in the N-586 HD flies, we performed seeding assays with or without PK treatment. Using equivalent amount of brains lysates as seeds, we find that the seeding activity of mutant HTT treated with PK is enhanced in flies colonized with *E. coli* ± curli (Figure 2). The elevated seeding activity is consistent with mobility defects and enhanced mortality of *E. coli-*treated N586-HD flies and suggest that gut bacteria may influence the production and/or the stability of seeding-competent and toxic HTT species.

Ubiquitously expressed full-length mutant HTT produces various abnormalities such as failure to elicit metamorphosis, cryptocephal phenotype, defects in abdominal and thoracic dorsal closure and rotated genitalia in the adult flies. These phenotypes are less severe when progenies are raised at low temperature (20°C) and this condition provides sufficient offspring with minimal or no visible structural defects. The progressive immobility of FL-HD flies is comparable between males and females (Supplementary Figure 4B). However, even at low temperature the rotated genitalia and abdominal cleft phenotype are more pronounced in males than in females. To avoid any compounding effects, which may arise from these and/or other undetected phenotypic and genotypic differences between the male and female HD flies, we selected a homogenous population of females with no visible structural pathology for the following studies.

**FIGURE 3:**
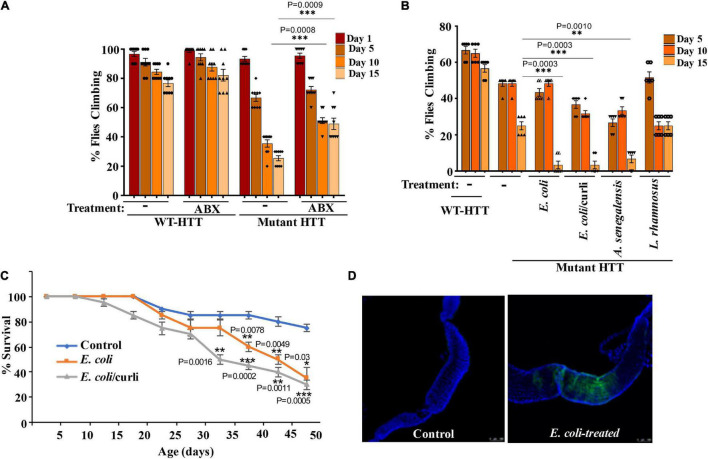
Gut bacteria regulate the motor function and lifespan of female FL-HD *Drosophila*. **(A)** Newly eclosed FL-HD flies or controls expressing full length HTT with 25Qs (WT-HTT) were treated with penicillin-streptomycin (ABX) for 15 days to eliminate gut bacteria. A climbing assay was performed at days 1, 5, 10, and 15 post-treatment. The data are graphed as mean ± SEM, two-way ANOVA with Tukey’s multiple-comparisons test. ***p < 0.001, *n* = 9 groups of 10 flies. **(B)** Bacteria-deficient adult FL-HD flies were fed different strains of *E. coli, A. senegalensis*, and *L. rhamnosus* for 15 days. Locomotive behavior for each condition was quantified as in part **(A)**. The data are reported as mean ± SEM, two-way ANOVA with Tukey’s multiple-comparisons test. ***p < 0.001; **p < 0.01, *n* = 6 groups of 10 flies. Part **(C)** shows the survival curve for FL-HD flies treated with two *E. coli* strains. The percent of flies that survived over time was calculated at different time points. The data are represented as mean ± SEM, two-way ANOVA with Tukey’s multiple-comparisons test. ***p < 0.001; **p < 0.01; *p < 0.05, *n* = 6 groups of 10 flies. **(D)** Immunostaining of the GI tract of control or *E. coli* (curli) treated FL-HD flies demonstrating bacterial colonization of fly gut. A monoclonal antibody generated to *E. coli* was used for detection. DAPI was used to stain the nuclei.

**FIGURE 4:**
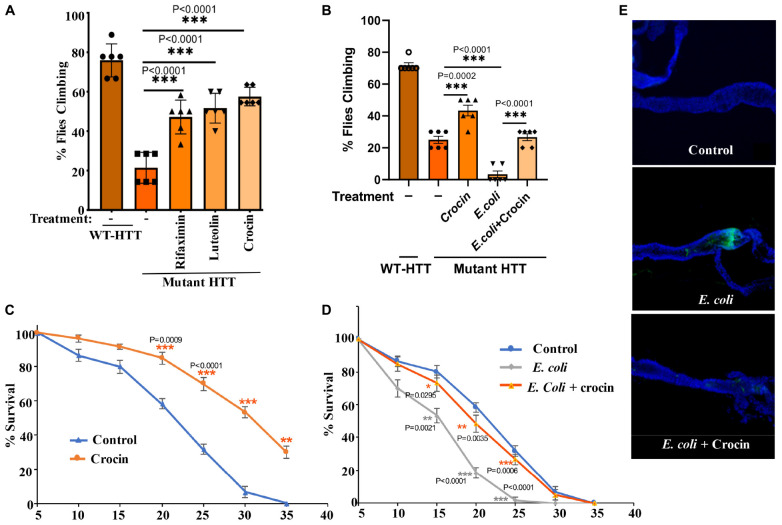
Crocin ameliorates *E. coli*-induced motor defects and mortality in female FL-HD flies. **(A)** Freshly eclosed FL-HD flies were treated with rifaximin, luteolin, and crocin for 15 days and their climbing ability was evaluated. Data are reported as mean ± SEM and were analyzed by one-way ANOVA with Tukey’s *post hoc* test. ***p < 0.001, *n* = 6 groups of 10 flies. **(B)** Climbing assay was performed to monitor the motor function of flies colonized with crocin, *E. coli* or *E. coli* plus crocin. Untreated FL-HD flies were used as control. Data are represented as mean ± SEM and were analyzed by one-way ANOVA with Tukey’s *post hoc* test. ***p < 0.001; **p < 0.01, *n* = 6 groups of 10 flies. Part **(C,D)** show the percentage of FL-HD flies, which survived over time (days) under different treatments. Temperature was elevated to 25°C to accommodate *E. coli* growth. The data are represented as mean ± SEM, two-way ANOVA with Tukey’s multiple-comparisons test. ***p < 0.001; **p < 0.01; *p < 0.05, *n* = 6 groups of 10 flies. **(E)** Representative confocal images of the GI tract of untreated (control) FL-HD flies or those treated with *E. coli* (curli) or *E. coli* plus crocin for 15 days showing bacterial colonization and suppression by crocin treatment. Immunostaining was performed using a monoclonal antibody reactive to *E. coli* DAPI was used to stain the nuclei.

To examine the role of gut bacteria on the mobility of FL-HD, we treated ∼3 day old adult female flies with rifaximin or penicillin-streptomycin and evaluated their climbing ability at different intervals. Antibiotic treatment ameliorates the motor defects of FL-HD flies (Figures 3A, 4A). Conversely, colonization of FL-HD flies with *E. coli* exacerbates the climbing defects but has no effects on the non-transgenic flies or those expressing full-length WT HTT (Figure 3B and Supplementary Figure 4A). Colonization of FL-HD or control flies with *L. rhamnosus* does not alter their climbing abilities. (Figure 3B and Supplementary Figure 4A). Notably, feeding excess *Acetobacter senegalensis* (*A. senegalensis*), a commensal gram-negative bacterium of *Drosophila* also accelerates the motor defects of FL-HD flies (Figure 3B). The similar debilitating climbing defects induced by two *E. coli* strains and *A. senegalensis* are consistent with a pathogenic role of gram-negative bacteria in the motor behavior of female HD flies. *E. coli* colonization also significantly reduces the lifespan of FL-HD flies and similar to the N-586 HD model, the curli-producing strain appears more pathogenic (Figure 3C). Immunostaining the gut of *E. coli*-fed female HD flies (FL-HD or the N-586 HD) shows colonization of *E. coli* in the midgut (Figure 3D and Supplementary Figure 5), which contains enterocytes and enteric neurons regulating host-microorganism interactions (Dutta et al., 2015; Capo et al., 2019). The localized accumulation of *E. coli* in the midgut is noteworthy as it confirms spatial colonization and a niche, which may participate in gut-brain communications in flies.

### Crocin Ameliorates Huntington’s Disease Phenotypes in Female *Drosophila* Models

**FIGURE 5:**
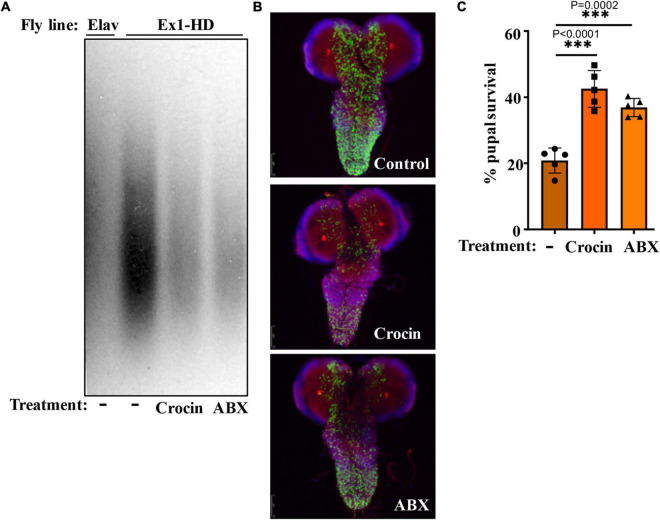
Crocin and antibiotics reduce HTTex1 aggregation and mortality. **(A)** SDD-AGE and WB analysis of lysates generated from the Ex1-HD larvae treated with crocin or penicillin/streptomycin (ABX). PHP1 antibody was used to detect aggregates. Elav-Gal4 larvae were used as a negative control. For each condition, ten larvae (*n* = 10) were pooled and analyzed. **(B)** Representative confocal images of brains from Ex1-HD larvae untreated (Control) and treated with crocin or ABX, and immunolabelled with PHP1 (green), anti-Elav antibody (red), and DAPI (blue). Part **(C)** shows the percent of pupal survival of Ex1-HD larvae treated with crocin or ABX. The data are represented as mean ± SEM, one-way ANOVA with Tukey’s *post hoc* test. *n* = 5 independent crosses for each condition, ***p < 0.001.

Gut-based regulation of mutant HTT neurotoxicity is a potential therapeutic target. Given that rifaximin treatment of Ex1-HD flies reduces the buildup of neurotoxic HTTex1 assemblies in neurons (Figures 1B,C), we examined whether gut-based natural compounds might have a similar effect. We focused on anti-inflammatory compounds since inflammation occurs early in pre-manifest HD patients and is recognized as a modifier of HD pathogenesis (Politis et al., 2015). Moreover, inflammation is a prominent outcome of dysbiosis linked to neurodegeneration (Sampson et al., 2016; Goyal et al., 2021). Toward this end, we generated Ex1-HD larvae on foods containing luteolin, a flavone which we have found to inhibit the IKKβ-dependent aggregation of HTTex1 in human neurons (Khoshnan et al., 2017), or crocin, a carotenoid abundant in the medicinal plant *Crocus sativus* (saffron), also known for its anti-inflammatory properties and inhibiting the aggregation of amyloidogenic proteins like α-synuclein (Inoue et al., 2018; Shafiee et al., 2018). We find that similar to antibiotics (rifaximin or penicillin-streptomycin), treatment with luteolin or crocin reduces the aggregation of HTTex1 in the nervous system of Ex1-HD larvae (Figures 1B,C, 5A,B, and Supplementary Figure 6). Crocin or antibiotics also enhance the eclosion efficiency of Ex1-HD flies (Figure 5C) further reinforcing gut-mediated regulation of HTTex1 aggregation, toxicity and survival.

Notably, addition of crocin, rifaximin or luteolin to fly food also ameliorates the motor defects of female FL-HD flies with crocin being the most potent compound (Figure 4A). Crocin also improves the severe motor defects of the FL-HD flies colonized with *E. coli* (Figure 4B). In survival assays, crocin extends the lifespan of female FL-HD flies and those treated with *E. coli* ± curli (Figures 4C,D, respectively). Mechanistically, we find that crocin treatment blocks the colonization and accumulation *E. coli* in the flies’ gut (Figure 4E and Supplementary Figure 7). Crocin does not inhibit the growth of E. *coli* in culture thus, its inhibitory effects on gut colonization may be due to the induction of flies’ anti-microbial defense pathways and/or altering the abundance and/or physiology of gut microbiota.

### Crocin Modulates the Composition of Gut Bacteria in Female Huntington’s Disease Flies

**FIGURE 6:**
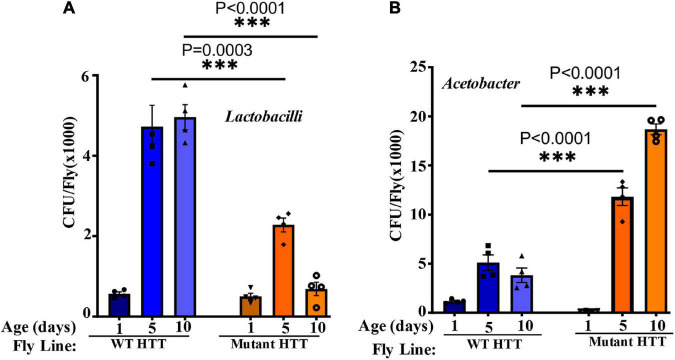
FL-HD female flies have lower abundance of *Lactobacilli* but elevated levels of *Acetobacter*. Microbial counts were determined by serial dilution plating of fly homogenates on MRS or Mannitol agar plates (section “Materials and Methods”). **(A,B)** Time course of the relative abundance of colony forming units (CFU) of *Lactobacilli* and *Acetobacter*, respectively. The data are represented as mean ± SEM, two-way ANOVA and Sidak’s multiple comparison Test. *n* = 4 agar plates for each condition, ^***^p < 0.001.

**FIGURE 7:**
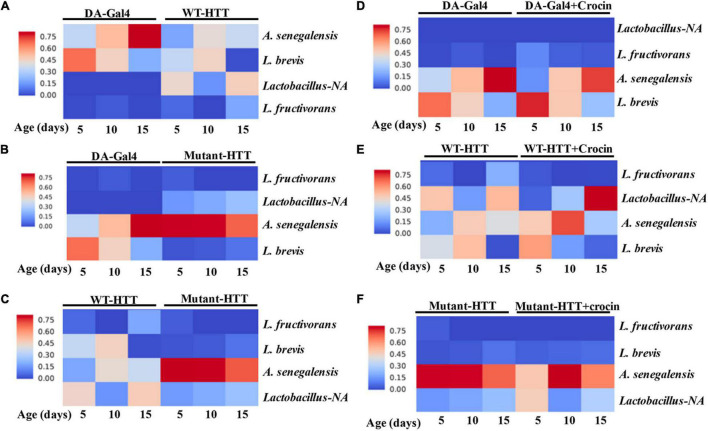
Relative abundance of *Lactobacilli* strains and *A. senegalensis* in the gut of female HD flies by 16S rRNA gene sequence analysis. **(A)** Comparative analysis of Da-Gal4 flies with those expressing WT-HTT, **(B)** Da-Gal4 with FL-HD flies, and **(C)** WT-HTT with FL-HD. Parts **(D–F)** show the effects of crocin on the abundance of bacteria in Da-Gal4, WT-HTT, and FL-HD flies, respectively. All analyses were performed as described in the section “Materials and Methods” by Zymogen’s computational biologists. *Lactobacillus-NA* strain was not detected in the data base.

We performed culture assays to examine any potential dysbiosis in the gut of female FL-HD flies. Notably, compared to flies expressing WT HTT, FL-HD flies begin to show dysbiosis by day 5 post eclosion exemplified by lower colony-forming units (CFUs) of *Lactobacilli* but elevated CFUs of *Acetobacter* (Figure 6). These findings are similar to results in Ex1-HD flies (Figure 1A and Supplementary Figure 1) and support an expanded polyQ-mediated disruption in the composition of gut bacteria. To gain better insights into the composition of gut bacteria and the impact of crocin, we performed 16S rRNA sequencing. Our *Drosophila* stocks including those with human HTT transgenes predominantly harbor several *Lactobacilli* strains and *Acetobacter senegalensis (A. senegalensis)* species. Notably, *Lactobacilli* and *Acetobacter* show temporal fluctuation in abundance independent of any transgenes, which may be related to flies’ physiology and nutritional demand (Schretter et al., 2018; Storelli et al., 2018; Henriques et al., 2020; Yamauchi et al., 2020; Ankrah et al., 2021) (Figures 7A–C and Supplementary Figure 8A). Flies ubiquitously expressing HTT transgenes (WT or mutant) differ with respect to the relative abundance of *Lactobacilli* and *A. senegalensis* when compared to control flies (Da-Gal4) (Figures 7A–C and Supplementary Figure 8A). Consistent with culture findings, female FL-HD flies harbor elevated levels of *Acetobacter* and changes in the abundance of various *Lactobacilli* species when compared to normal flies or those expressing WT HTT (Figure 7C and Supplementary Figure 8A). Interestingly, elevation of *Acetobacter* has been linked to induced mortality in *Drosophila* (Obata et al., 2018). Indeed, female HD flies fed excess *A. senegalensis* develop severe motor defects, which are similar in magnitude to those induced by *E. coli* (Figure 3B). These data are consistent with the induction of dysbiosis by mutant HTT in *Drosophila* and a potential link to disease progression. Crocin treatment alters the abundance of *Acetotbacter* and *Lactobacillus* in all tested fly lines independent of HTT transgene expression (Figures 7D–F and Supplementary Figure 8B). Crocin also reduces the elevated levels of *Acetobacter* but increases the abundance of *Lactobacillus-NA* (not annotated in data bank) in 5-day old female FL-HD flies, however, the effects do not persist (Figure 7F and Supplementary Figure 8B). We predict that crocin may alter the signaling pathways, which regulate the abundance and/or the physiology of *Lactobacilli* and *Acetobacter* species based on the flies’ nutritional demand (Storelli et al., 2018; Yamauchi et al., 2020). Such systemic and complex metabolic changes may translate into protection observed in the crocin-treated HD flies and remain to be investigated at a molecular level.

## Discussion

We took advantage of the simplicity of microbiota in *Drosophila* to reveal how bacteria may affect the development of HD. We provide evidence that gut bacteria promote mutant HTT aggregation, impair locomotion and reduce the lifespan of transgenic female HD flies. More specifically, we have identified the gram-negative *Acetobacter*, a commensal fly bacterium, and *E. coli* a human pathobiont, as modifiers of HD pathogenesis. As a proof of concept, we have further demonstrated that modifying the gut environment of HD flies by a gut-specific antibiotic rifaximin, which is approved to treat dysbiosis in Hepatic Encephalopathy patients (Bureau et al., 2021), or small nutraceuticals such as luteolin or crocin delays HD progression. These studies are a prelude to future mechanistic investigations of microbiota-brain interactions in HD and identification of pathway, which may serve as targets for gut-based therapeutic interventions. A limitation of the current study however, is the use of just female flies in behavioral studies. While both sexes display HD phenotypes including HTT aggregation and immobility, the response of male *Drosophila* expressing mutant HTT to microbial and dietary manipulations of the gut environment may differ from females and remains to be investigated.

Our results indicate that elimination of gut bacteria by antibiotics in the Ex1-HD flies significantly reduces the aggregation of amyloidogenic N-terminal HTTex1. These findings are further reinforced by the induction of mutant HTT aggregation by two strains of *E. coli* in the N-586 HD flies. Interestingly, *E. coli* colonization elevated the seeding activity of mutant HTT (Figure 2), which is linked to severity of symptoms in HD animal models and neurotoxicity in human neurons (Ast et al., 2018; Chongtham et al., 2021). The notion that gut bacteria may alter the toxicity of mutant HTT assemblies is a novel finding, which requires biophysical investigation to identify any potential neurotoxic conformations (Ko et al., 2018; Chongtham et al., 2021). Recent studies indicate that gram-negative bacteria promote the aggregation of polyQ peptides and impair the motility of *Caenorhabditis elegans* by disrupting proteostasis in neurons, muscle and intestinal cells (Walker et al., 2021). Our studies are consistent with these findings and underscore the regulatory role of gut bacteria in promoting the misfolding and aggregation of amyloidogenic HTT fragments and the production of neurotoxic assemblies. Surprisingly, several bacterial species have been detected in the brain tissues of HD patients. Among them are members of *Enterobacteriaceae* family, which include *E. coli* and other gram-negative bacteria (Alonso et al., 2019). How bacteria gain access to the brains of HD patients and whether they affect the proteostasis of HTT remains unknown. Our results however, indicate that bacteria such as *E. coli* may promote the aggregation of HTT from the gut. Notably, antibiotic treatments of Ex1-HD flies (expressing mutant HTTex1 selectively in neurons) reduce the aggregation of HTTex1 (Figures 1B,C, 5A,B) thus, supporting an indirect role for gut microbiota regulating the aggregation of amyloidogenic HTT fragments in the nervous system. Ex1-HD flies also show signs of gut dysbiosis (Figure 1A). Given that enteric neurons directly interact with the intestinal epithelium, we speculate that mutant HTTex1 may disrupt neuronal signals essential for gut microbial homeostasis. While details remain to be understood, the findings support the notion that gut dysbiosis in HD flies may also be triggered by neuronal defects. Collectively, we predict that aberrant bidirectional signaling may feed one another and contribute to gut-brain dysfunction in HD.

*E. coli* expressing the functional amyloids curli in the gut has been implicated in α-synuclein aggregation in the brains of rodents, which coincides with PD pathology (Chen et al., 2016; Sampson et al., 2020). Our data did not show a selective impact of curli-producing *E. coli* on HTT aggregation and impaired locomotion of female HD flies but curli had a negative effect on lifespan and promoted HTTex1 aggregation when co-expressed in mammalian tissue culture (Figures 1, 3 and Supplementary Figure 2). These findings suggest that curli amyloids may need close contact with HTT to promote its aggregation and that they are potentially unable to enter *Drosophila* cells. Moreover, the negative effects of curli on the survival of female HD flies may be independent of HTT aggregation. Notably, *E. coli* and elevated *Acetobacter* (a fly commensal) produced similar debilitating effects on locomotion, which is consistent with gram-negative bacteria being modifiers of HD symptoms. A recent study demonstrated a correlation between the abundance of the gram-negative *Bilophila* species and elevated inflammatory cytokine response in HD patients (Du et al., 2021). Our findings along with studies on the microbiota of HD patients and mammalian models support the potential involvement of specific clades (or a specific clade) of gut bacteria regulating HD progression and severity (Kong et al., 2020; Stan et al., 2020; Wasser et al., 2020; Du et al., 2021; Kong et al., 2021).

The ability to modify HD pathology from the gut is therapeutically useful and attractive. Inclusion of crocin in the diet suppressed HTT aggregation, ameliorated locomotive defects, and increased the survival of female HD flies including those colonized with *E. coli.* The effects of crocin on the diversity of gut bacteria in *Drosophila* were difficult to dissect due to few commensal species. However, crocin treatment altered the abundance of *Lactobacillus* and *Acetobacter* in the young female FL-HD flies (Figure 7F) and prevented *E. coli* colonization in the gut (Figure 4E and Supplementary Figure 7). In rodents, crocin modifies gut microbiota, which coincides with the inhibition of neurodegeneration in cerebral ischemia models (Zhang et al., 2019), and mitigates glucocorticoids-induced dysbiosis in mice by increasing the alpha diversity of gut bacteria and subsequent normalization of aberrant lipid metabolism (Xie et al., 2019). Crocin and its major byproduct crocetin do not penetrate the gut epithelium in mammals suggesting that its protective effects are potentially induced by modification of gut physiology and/or its microbiota (Xie et al., 2019; Zhang et al., 2019). Given that alpha diversity of gut bacteria is decreased in HD patients and in HD mice (Kong et al., 2020; Stan et al., 2020; Wasser et al., 2020; Du et al., 2021), the application of crocin as a promoter of bacterial diversity in HD models with complex microbiota may provide useful knowledge on specific species, which regulate HD progression.

In summary, our data support a link between gut bacteria and disease progression in transgenic *Drosophila* models of HD and highlight the involvement of gram-negative bacteria in regulating the abundance and toxicity of mutant HTT. The neurotoxic phenotypes such as protein aggregation, motor defects and mortality induced by *E. coli* are useful biomarkers to investigate the gut-brain circuits at a cellular and molecular level. The *Drosophila* GI tract shares anatomical and functional similarities to the mammalian equivalent (Apidianakis and Rahme, 2011). The midgut where *E. coli* colonizes (Figures 3 and 5) is enriched in numerous cell types including the enteroendocrine (EE) cells, which perform similar tasks as in human GI cells and are nodes for gut-brain networks implicated in neurodegeneration (Chandra et al., 2017). Future knowledge on the transcriptome and proteomics of EE cells in HD *Drosophila* models may identify some of the earliest events, which may trigger the neurotoxicity of mutant HTT in the nervous system. The inclusion of crocin as an inhibitory agent of *E. coli* toxicity may also identify overlapping pathways, potentially functioning in the opposite directions, or compensatory circuits beneficial to HD. Crocin has a long history of medicinal use and has produced favorable outcomes in clinical and preclinical trials in CNS disorders including AD, PD, ischemia, mood disorders, and LPS-induced cognitive decline (Rajaei et al., 2016; Zhang et al., 2018; Haeri et al., 2019; Zhang et al., 2019; Azmand and Rajaei, 2021). The therapeutic benefits of crocin in subjects with depression have been attributed to the induction of neurortrophin expression including brain-derived neurotropic factor (BDNF) (Ghasemi et al., 2015; Moghadam et al., 2021). This property of crocin may prove useful for HD considering the role of HTT in BDNF expression and transport, biological activities that are diminished in neurons expressing mutant HTT (Zuccato and Cattaneo, 2014). These features and the gut-modifying properties of crocin make it or its derivatives attractive therapeutic candidates for HD.

## Data Availability Statement

The original contributions presented in this study are included in the article/Supplementary Material. Further inquiries can be directed to the corresponding author/s.

## Author Contributions

AK conceived the idea, wrote the manuscript, and acquired the funding. AC contributed to writing and editing the manuscript. AC, JY, TC, NA, and AH performed the experiments. AK, AC, and JY analyzed the data. JM provided the fly stocks. All authors have read and approved the manuscript.

## Conflict of Interest

The authors declare that the research was conducted in the absence of any commercial or financial relationships that could be construed as a potential conflict of interest.

## Publisher’s Note

All claims expressed in this article are solely those of the authors and do not necessarily represent those of their affiliated organizations, or those of the publisher, the editors and the reviewers. Any product that may be evaluated in this article, or claim that may be made by its manufacturer, is not guaranteed or endorsed by the publisher.
