# Corrigendum: Gut bacteria regulate the pathogenesis of Huntington's disease in *Drosophila* model

**DOI:** 10.3389/fnins.2022.991513

**Published:** 2022-10-13

**Authors:** Anjalika Chongtham, Jung Hyun Yoo, Theodore M. Chin, Ngozi D. Akingbesote, Ainul Huda, J. Lawrence Marsh, Ali Khoshnan

**Affiliations:** ^1^Biology and Bioengineering, California Institute of Technology (Caltech), Pasadena, CA, United States; ^2^Developmental and Cell Biology, University of California, Irvine, Irvine, CA, United States

**Keywords:** Huntington's disease, microbiota, gut-brain, neurodegeneration, crocin (PubChem CID: 5281233)

In the published article, there was an error in Figure 4 as published. We inadvertently duplicated Figure 4A, instead of Figure 4B. The correct figure and its caption appear below.

**Figure 4:**
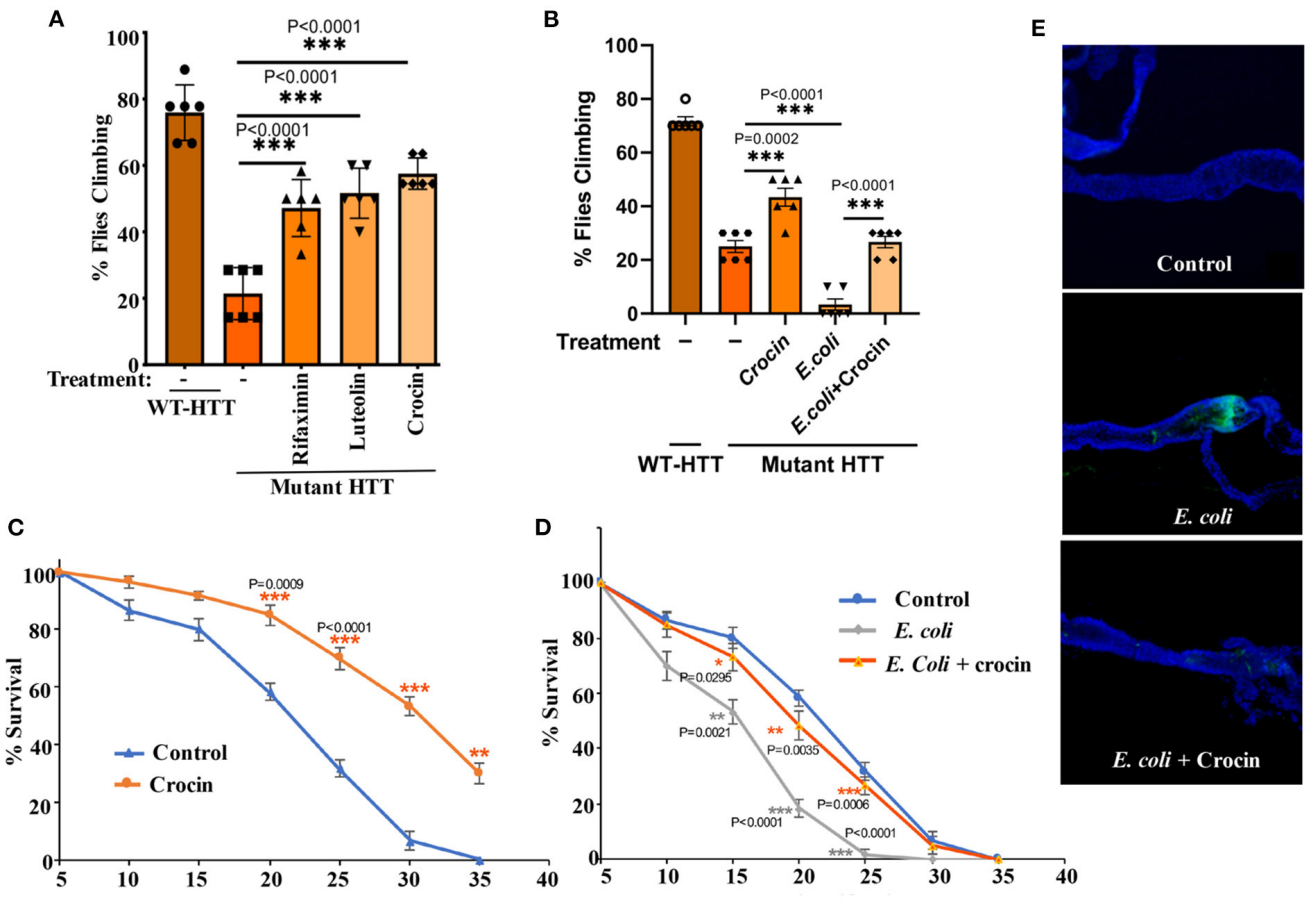
Crocin ameliorates *E. coli*-induced motor defects and mortality in female FL-HD flies. **(A)** Freshly eclosed FL-HD flies were treated with rifaximin, luteolin, and crocin for 15 days and their climbing ability was evaluated. Data are reported as mean ± SEM and were analyzed by one-way ANOVA with Tukey's *post hoc* test. ***p < 0.001, *n* = 6 groups of 10 flies. **(B)** Climbing assay was performed to monitor the motor function of flies colonized with crocin, *E. coli* or *E. coli* plus crocin. Untreated FL-HD flies were used as control. Data are represented as mean ± SEM and were analyzed by one-way ANOVA with Tukey's *post hoc* test. ***p < 0.001; **p < 0.01, *n* = 6 groups of 10 flies. Part **(C,D)** show the percentage of FL-HD flies, which survived over time (days) under different treatments. Temperature was elevated to 25°C to accommodate *E. coli* growth. The data are represented as mean ± SEM, two-way ANOVA with Tukey's multiple-comparisons test. ***p < 0.001; **p < 0.01; *p < 0.05, *n* = 6 groups of 10 flies. **(E)** Representative confocal images of the GI tract of untreated (control) FL-HD flies or those treated with *E. coli* (curli) or *E. coli* plus crocin for 15 days showing bacterial colonization and suppression by crocin treatment. Immunostaining was performed using a monoclonal antibody reactive to *E. coli*. DAPI was used to stain the nuclei.

The authors apologize for this error and state that this does not change the scientific conclusions of the article in any way. The original article has been updated.

## Publisher's note

All claims expressed in this article are solely those of the authors and do not necessarily represent those of their affiliated organizations, or those of the publisher, the editors and the reviewers. Any product that may be evaluated in this article, or claim that may be made by its manufacturer, is not guaranteed or endorsed by the publisher.

