# Supplementary figures and images for: Gut Bacteria Regulate the Pathogenesis of Huntington’s Disease in Drosophila Model
Source: Front Neurosci. 2022 Jun 2;16:902205. doi: 10.3389/fnins.2022.902205 (PMC9215115; doi:10.3389/fnins.2022.902205)

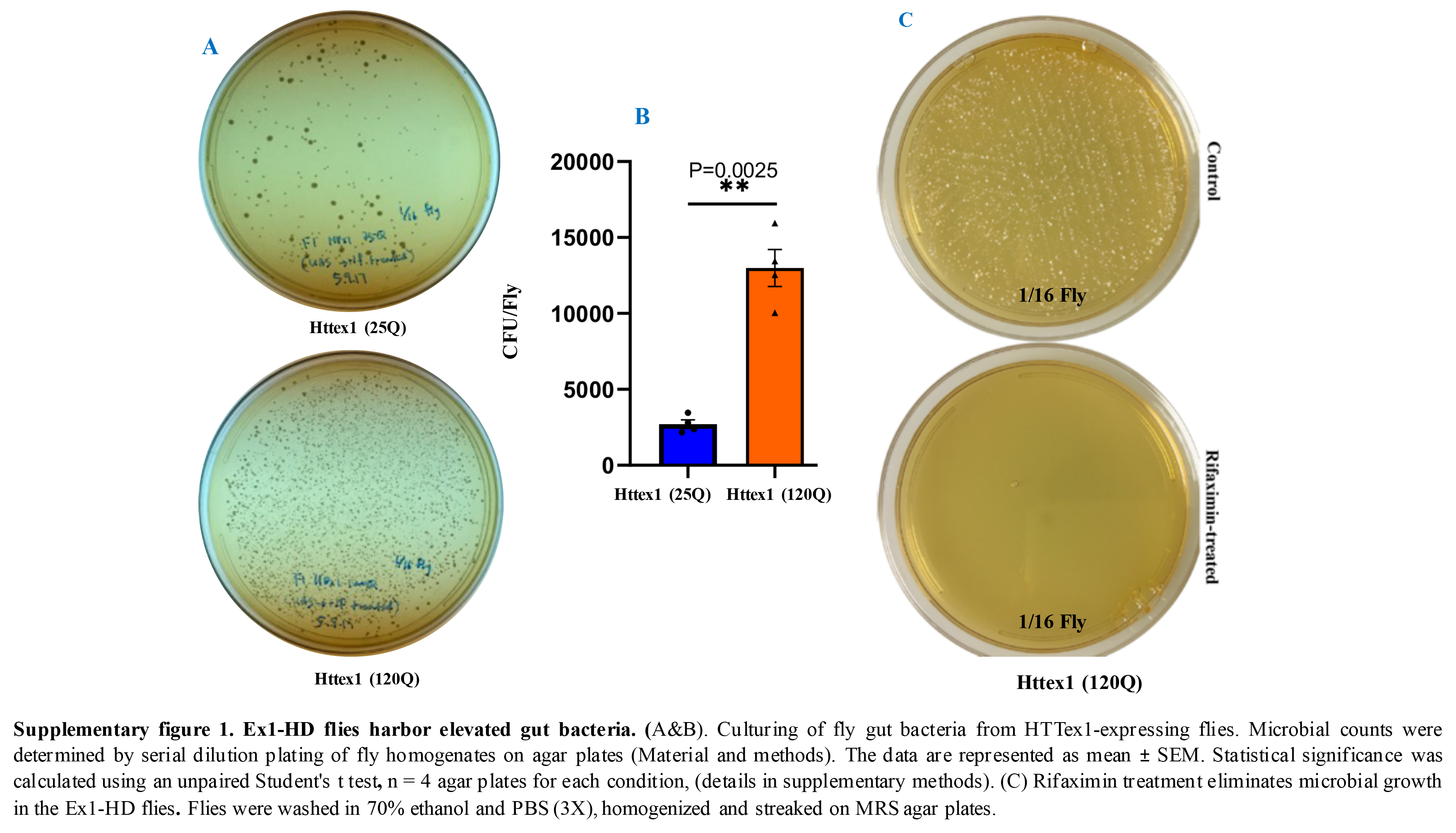

Supplement: Supplementary file 1 [file Data_Sheet_1.ZIP › Suppl. 1.tif]

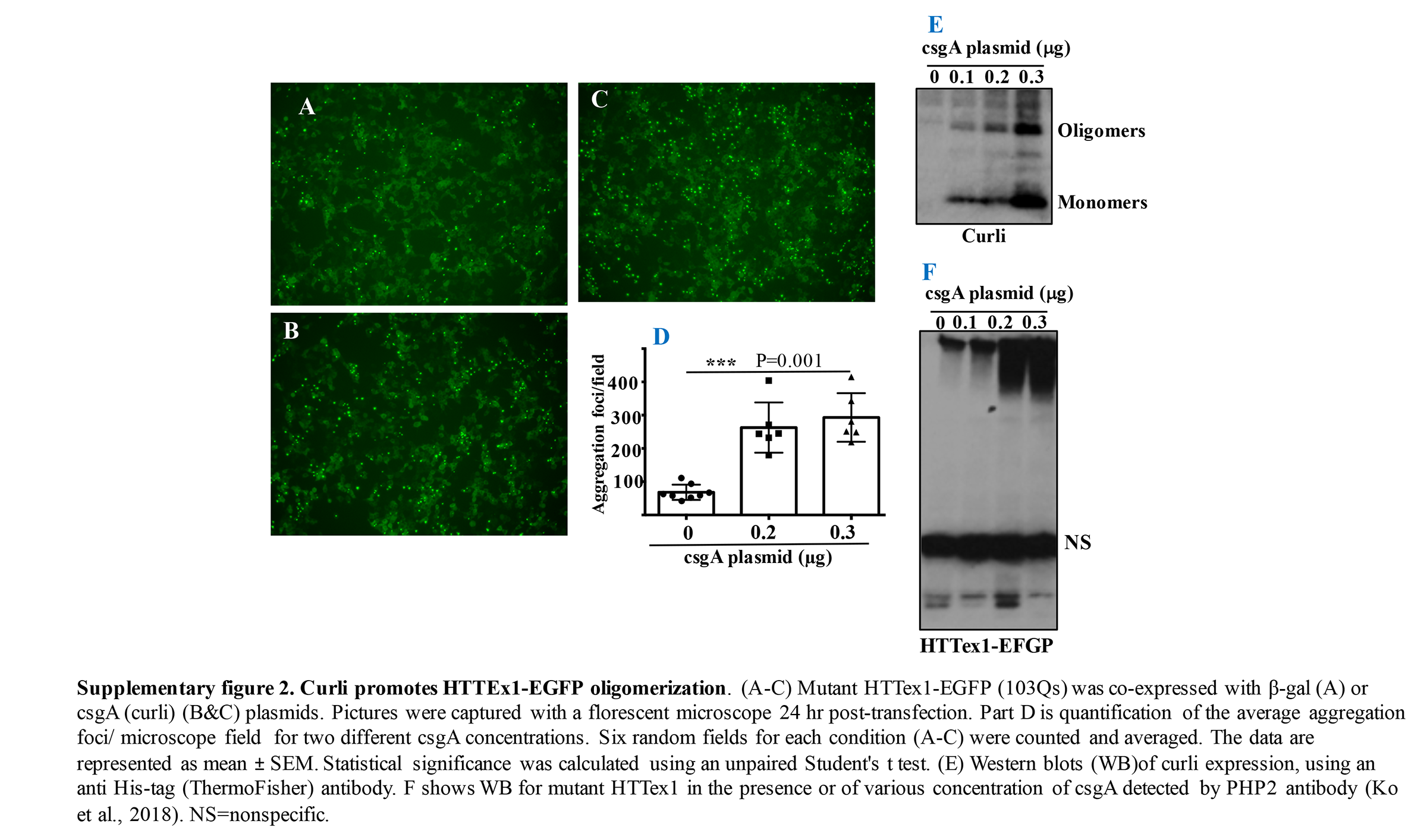

Supplement: Supplementary file 1 [file Data_Sheet_1.ZIP › Suppl. 2.tif]

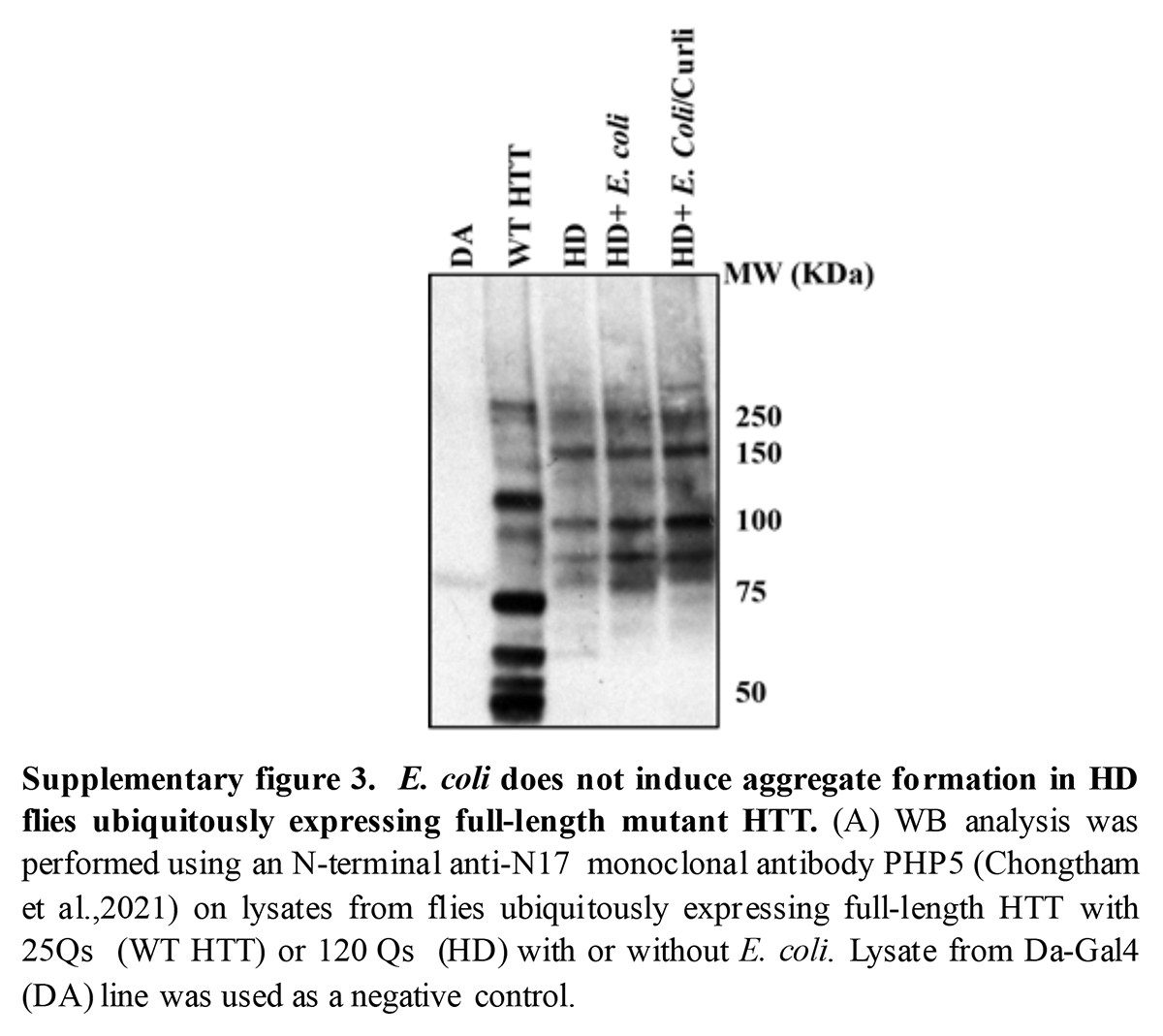

Supplement: Supplementary file 1 [file Data_Sheet_1.ZIP › Suppl. 3.tif]

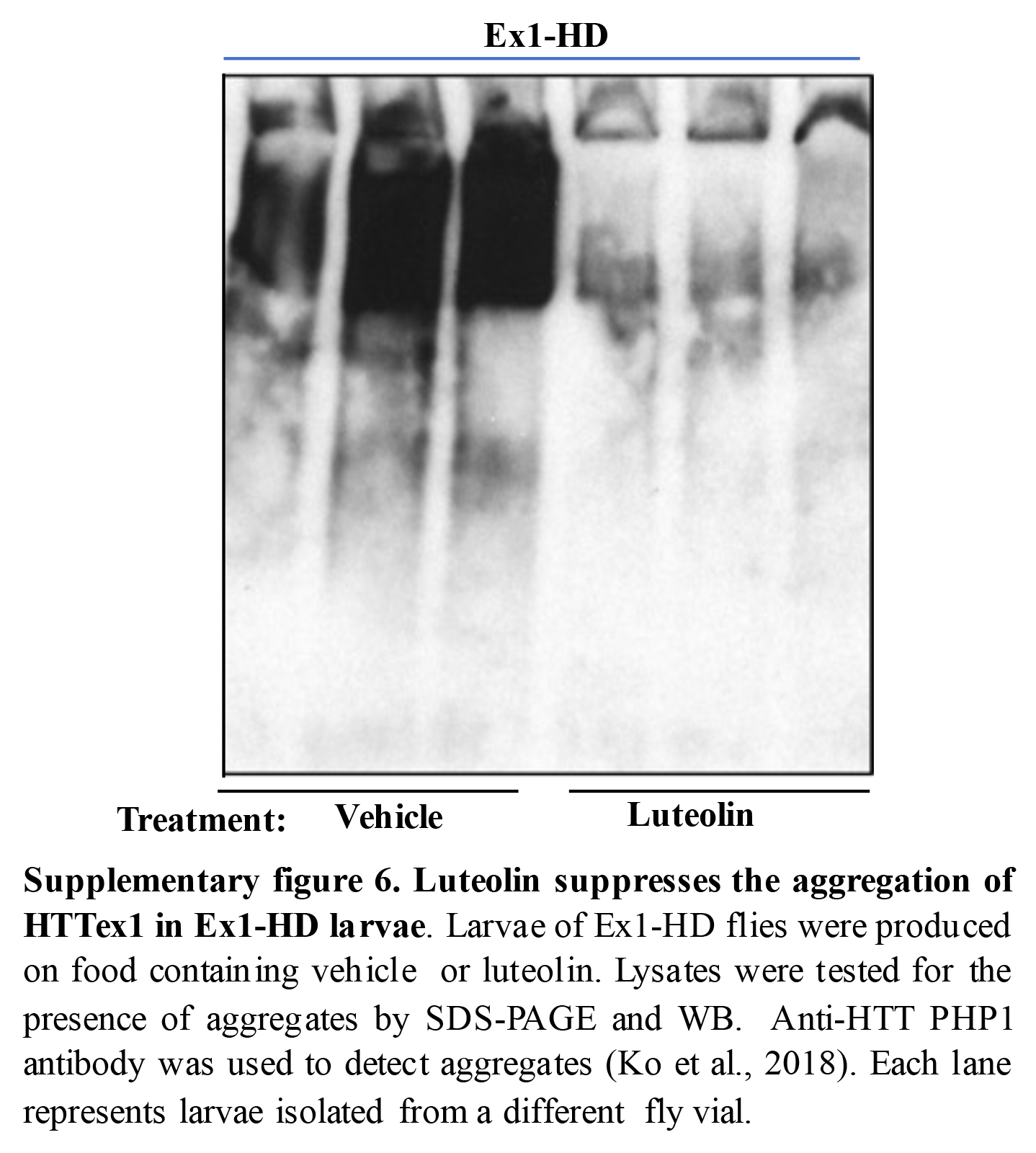

Supplement: Supplementary file 1 [file Data_Sheet_1.ZIP › Suppl. 6.tif]

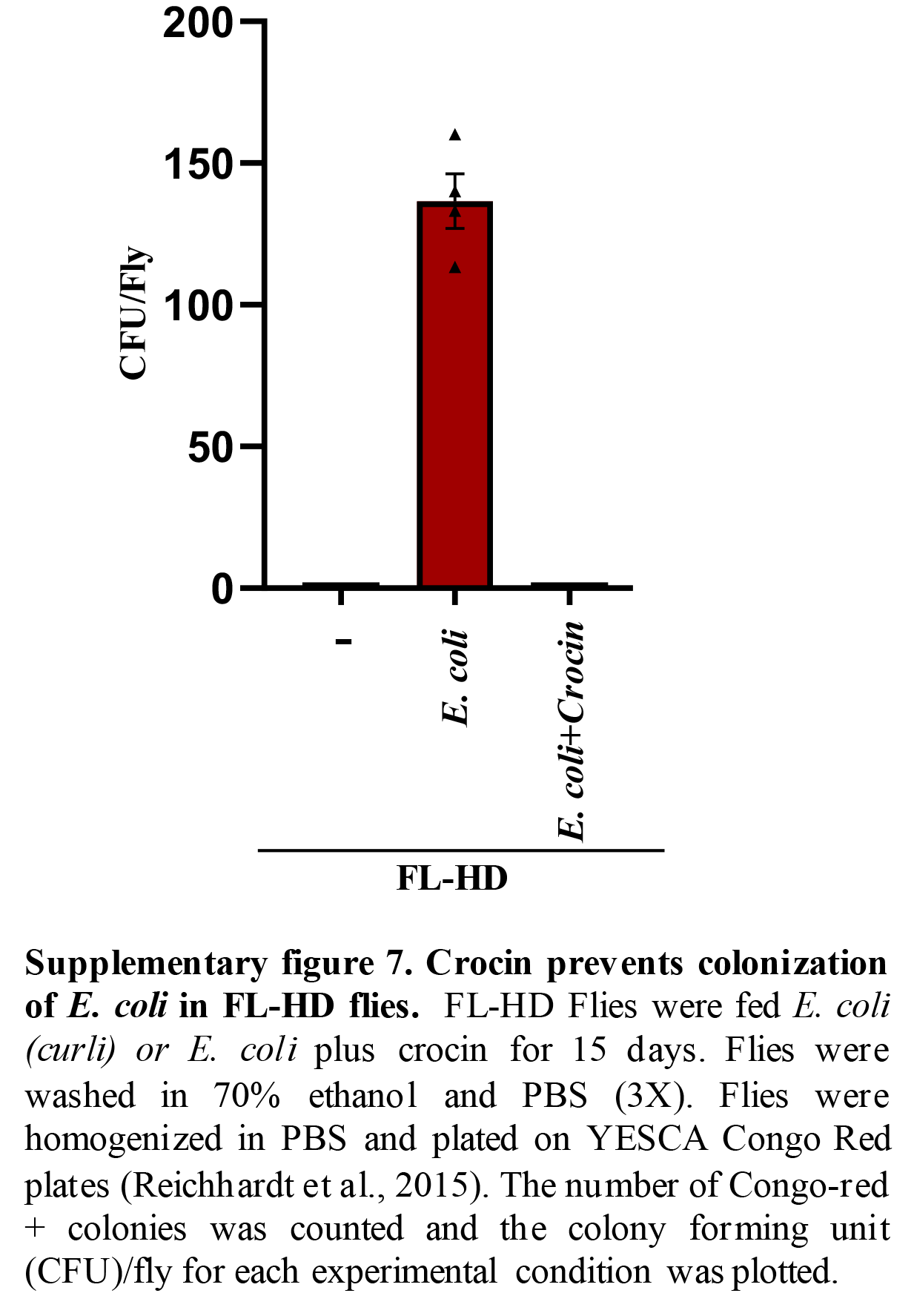

Supplement: Supplementary file 1 [file Data_Sheet_1.ZIP › Suppl. 7.tif]

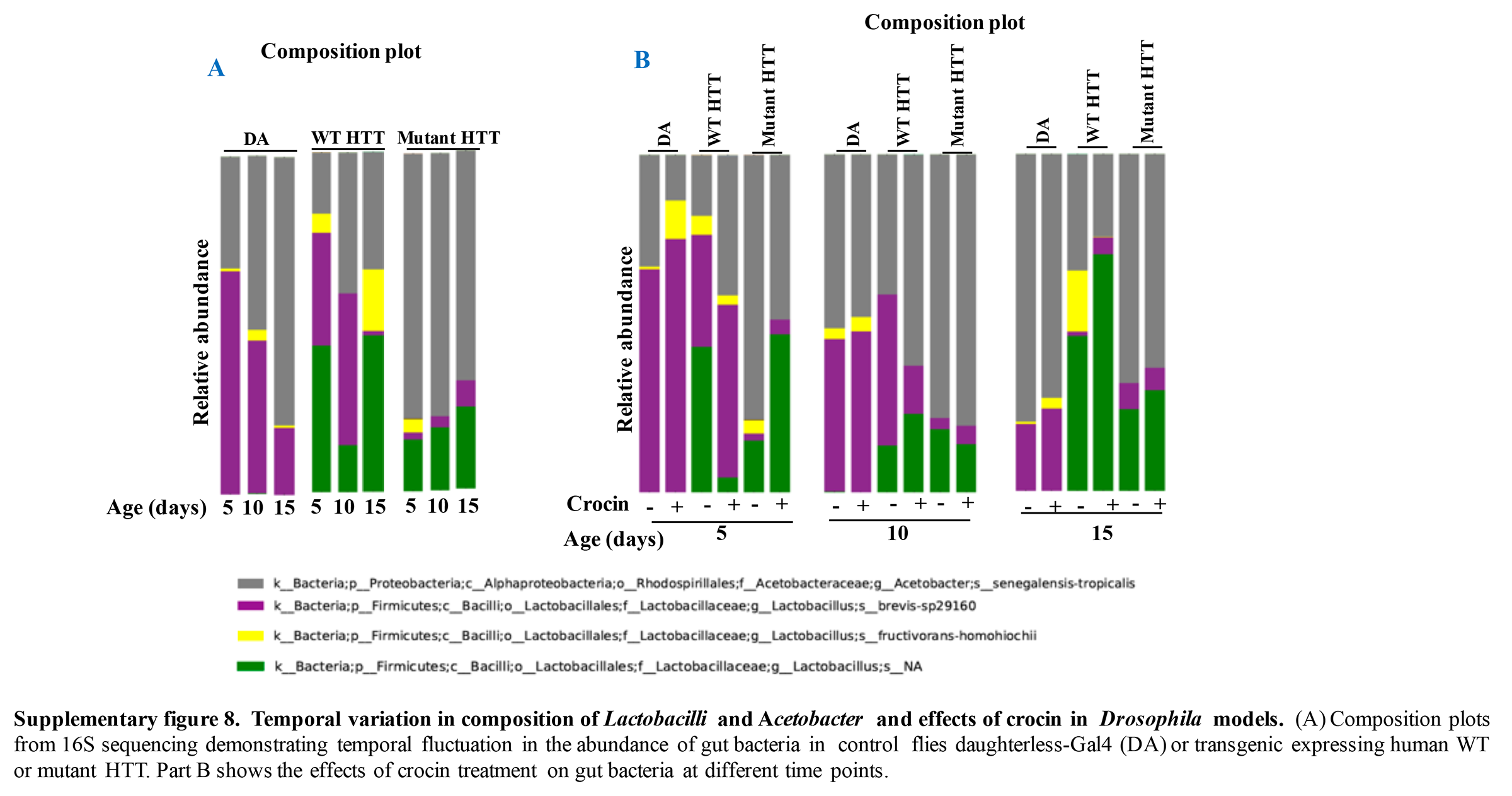

Supplement: Supplementary file 1 [file Data_Sheet_1.ZIP › Suppl. 8.tif]

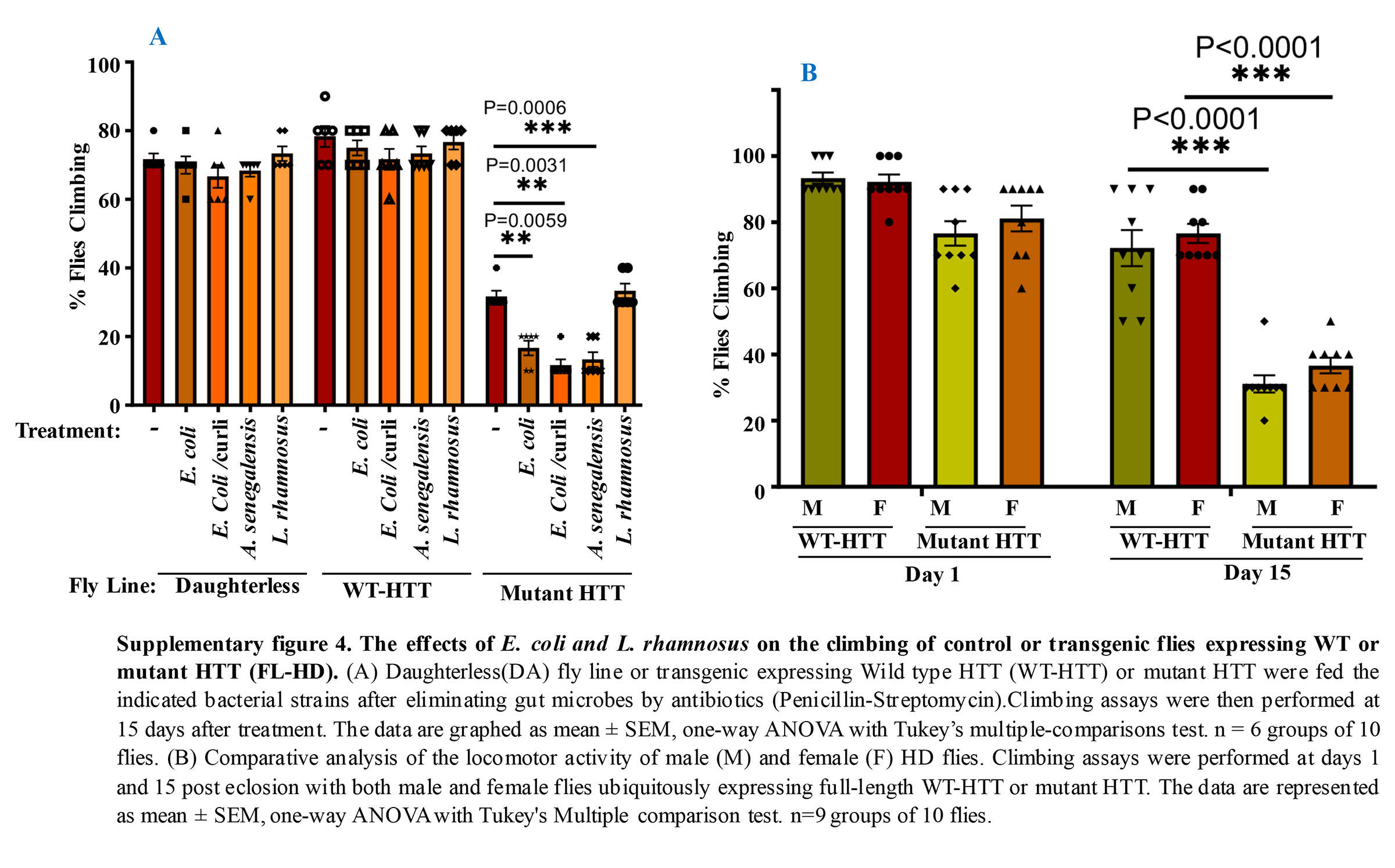

Supplement: Supplementary file 1 [file Data_Sheet_1.ZIP › Suppl.4.tif]

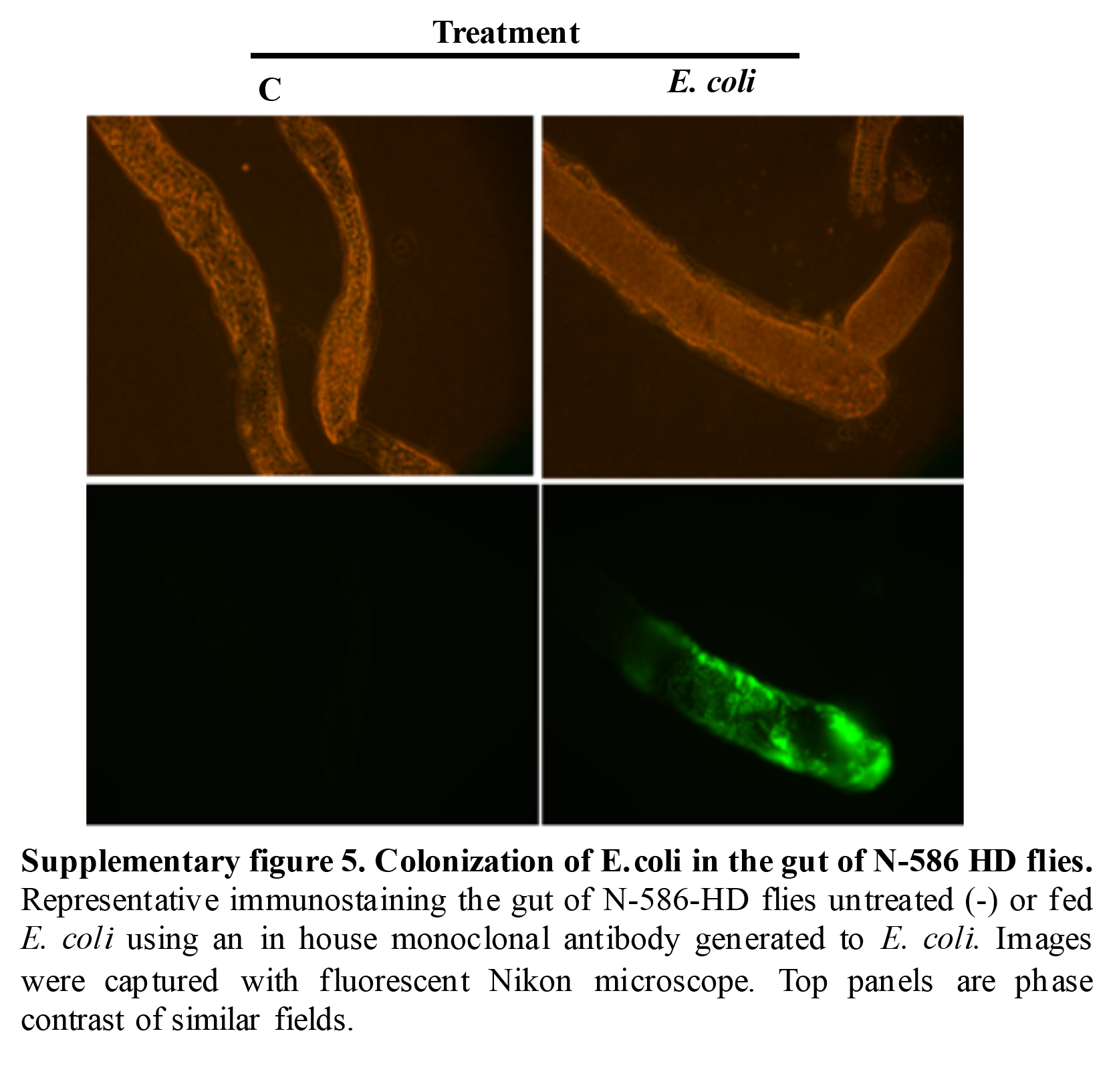

Supplement: Supplementary file 1 [file Data_Sheet_1.ZIP › Suppl.5.tif]
